# Diacerein ameliorates thioacetamide-induced hepatic encephalopathy in rats via modulation of TLR4/AQP4/MMP-9 axis

**DOI:** 10.1007/s11011-024-01457-x

**Published:** 2024-11-18

**Authors:** Nesma A. Abd Elrazik, Al Shaima G. Abd El Salam

**Affiliations:** Department of Biochemistry, Faculty of Pharmacy, Mansoura University, Mansoura, 35516 Egypt

**Keywords:** Hepatic encephalopathy, Diacerein, TLR4, Aquaporin 4, GFAP, MMP-9

## Abstract

Astrocyte swelling, blood brain barrier (BBB) dissipation and the subsequent brain edema are serious consequences of persistent hyperammonemia in hepatic encephalopathy (HE) in which if inadequately controlled it will lead to brain death. The current study highlights the potential neuroprotective effect of diacerein against thioacetamide (TAA)-induced HE in acute liver failure rat model. HE was induced in male Sprague–Dawley rats via I.P. injection of TAA (200 mg/kg) for three alternative times/week at 3^rd^ week of the experiment. Diacerein (50 mg/kg) was gavaged for 14 days prior to induction of HE and for further 7 days together with TAA injection for an overall period of 21 days. Diacerein attenuated TAA-induced HE in acute liver failure rat model; as proofed by significant lowering of serum and brain ammonia concentrations, serum AST and ALT activities and significant attenuation of both brain and hepatic MDA contents and IL-1β with marked increases in GSH contents (*P* < 0.0001). The neuroprotective effect of diacerein was demonstrated by marked improvement of motor and cognitive deficits, brain histopathological changes; hallmarks of HE. As shown by immunohistochemical results, diacerein markedly downregulated brain TLR4 expression which in turn significantly increased the GFAP expression, and significantly decreased AQP4 expression; the astrocytes swelling biomarkers (*P* < 0.0001). Moreover, diacerein preserved BBB integrity via downregulation of MMP-9 mediated digestion of tight junction proteins such as occludin (*P* < 0.0001). Collectively, diacerein ameliorated cerebral edema and maintained BBB integrity via modulation of TLR4/AQP4/MMP-9 axis thus may decrease the progression of HE induced in acute liver failure.

## Introduction

Hepatic encephalopathy (HE) is a dysfunction in the brain due to liver insufficiency, which includes a broad spectrum of neuropsychiatric abnormalities like impaired memory, shortened attention, sleep disturbances, psychomotor dysfunction and cognitive abnormalities which may reach ultimately to coma (Hussien et al. 2022). HE leads to repeated hospitalizations, impairing life quality and increased rates of death (Agrawal et al. 2015).

For induction of acute liver failure and HE in experimental animals, thioacetamide (TAA) is used owing to its good reproducibility and well-described liver and brain changes which similar to that occur in human (Lima et al. 2019). TAA is metabolized by cytochrome P450 to TAA-S-oxide which in turn stimulates generation of reactive oxygen species and hyperammonemia (Sepehrinezhad et al. 2021).

During HE, deterioration in liver function leads to elevation in blood ammonia level that crosses the blood-brain barrier (BBB) (Suraweera et al. 2016). Hyperammonemia induces oxidative stress and releases the pro-inflammatory mediators *via* activation of toll-like receptor-4 (TLR4). The detoxification of ammonia in astrocytes and TLR4 stimulation result in excess water entry, astrocytes swelling and finally brain edema (El-Baz et al. 2021). Moreover, ammonia and oxidative stress can cause swelling of astrocytes through increasing in aquaporin 4 (AQP4) water channel expression accompanied with entrance of water into cells (Sepehrinezhad et al. 2020).

Additionally, the neuro-inflammation leads to increase in brain matrix metalloproteinase 9 (MMP-9) level that digest tight junction proteins such as occludin causing apparent disruption in BBB integrity and hence cerebral edema (Dhanda and Sandhir 2018; Sepehrinezhad et al. 2020; Abo El gheit et al. 2020).

Decreasing inflammation has a beneficial therapeutic effect in curing cognitive dysfunction in HE (Lu et al. 2021). Diacerein, an anthraquinone derivative, is approved for osteoarthritis treatment. It possess a wide range of pharmacological activities involving anti-inflammatory and antioxidant effects (Abd-Ellatif et al. 2019; Almezgagi et al. 2020). The anti-inflammatory activity of diacerein is attributed to its capability to inhibit the production of inflammatory cytokines such as interleukin-1 beta (IL-1β) and blocks its receptors, as well as the down-regulation of TLR4 signaling pathway activation (Ibrahim et al. 2021; Ali et al. 2023). Moreover, it has been reported that diacerein has a protective effect against acetaminophen hepatotoxicity and liver ischemia/reperfusion damage (Ibrahim et al. 2021; Ali et al. 2023; Mohamed Kamel et al. 2022). However, its effect on HE induced by TAA remains unclear.

Thus, this study assessed the potential neuroprotective effect of diacerein on TAA-induced HE in rats and investigated its impact on brain edema and astrocyte swelling through TLR4 mediated inflammation, AQP4 channels and BBB integrity.

## Materials and methods

###  Chemicals

**TAA** was purchased from Sigma–Aldrich (St. Louis, MO, USA) and it was dissolved in 0.9% (w/v) NaCl for intraperitoneal (I.P.) administration.

**Diacerein** was purchased from Eva Pharma Company (Cairo, Egypt) and it was suspended in 0.5% (w/v) carboxymethylcellulose (CMC) for oral administration.

### Animals and ethical statement

Forty-eight male Sprague–Dawley rats (7–8 weeks age weighing 160–200 g) were purchased from the Egyptian Organization for Biological Products and Vaccines (Giza, Egypt) and were kept under standard conditions (45–55% humidity, temperature 25 ± 2 ^◦^C, 12 h light/dark cycle, with free access to food and water). The experimental protocol was carried out in accordance with the Animal Care and Use Committee of Mansoura University (MU-ACUC), Egypt; MU-ACUC (PHARM.R.23.04.16), complied with “Guide for the Care and Use of Laboratory Animals” (National Research Council publication 8th ed, USA, 2011).

### Experimental design and sample collection

Induction of HE has been done by I.P. injection of TAA (200 mg/kg) three times/week at three alternative days during the third week of the experiment. The duration of the experimental model was 21 days (Khodir and Said 2020).

**Fig. 1:**
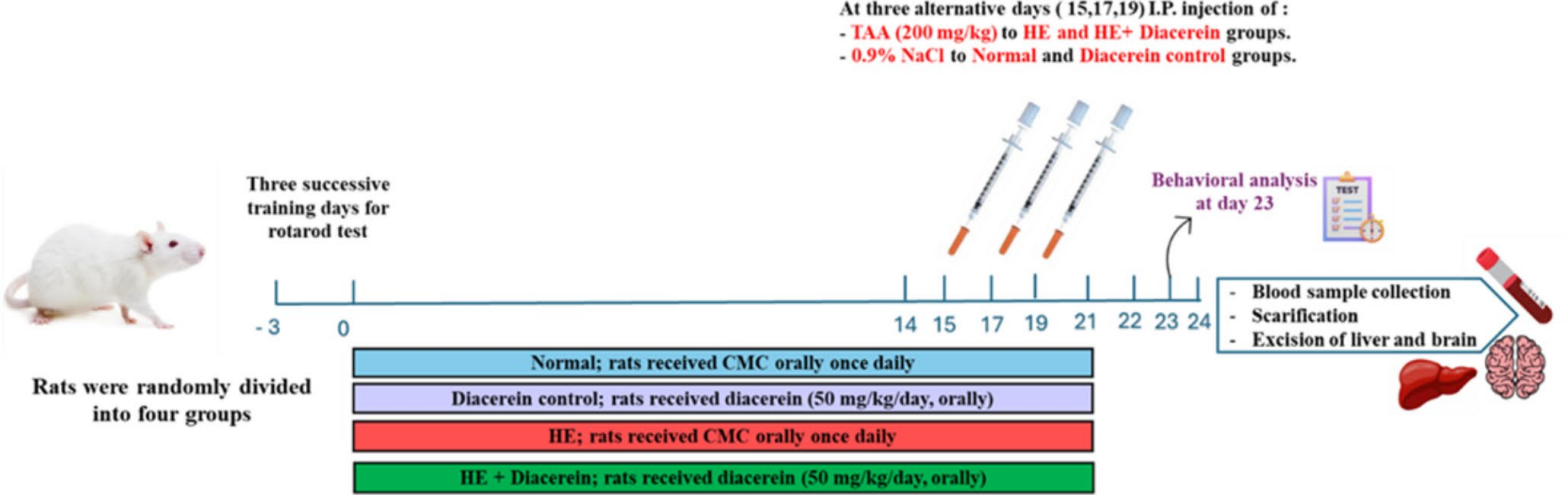
Schematic representation of the experimental design

Rats were randomly divided into four groups (*n* = 12/group) as follows: Normal; rats received CMC orally once daily for two weeks before first 0.9% NaCl injection and then 0.9% NaCl was injected 3 times in alternative days along with daily CMC administration on the third week of the experiment. Diacerein control; rats received diacerein (50 mg/kg/day, oral) (Ibrahim et al. 2022) once daily for two weeks before first 0.9% NaCl injection and then 0.9% NaCl was injected 3 times in alternative days along with daily diacerein administration on the third week of the experiment. HE; rats received the CMC orally/day for two weeks before first TAA injection and then TAA was injected 3 times in alternative days along with daily CMC administration on the third week of the experiment. HE + Diacerein; rats received diacerein (50 mg/kg/day, oral) (Ibrahim et al. 2022) for two weeks before first TAA injection and then TAA was injected 3 times in alternative days along with daily diacerein administration on the third week of the experiment (Fig. 1).

The started number of rats in HE group was 14 rats, and 2 rats died during the experiment.

To prevent TAA-induced hypoglycemia and electrolyte imbalance, rats were injected subcutaneously with a solution containing 10% dextrose water mixed with a lactate ringer solution (25 mL/kg) every 12-hr. following the first injection of TAA (Baraka et al. 2020). Six rats from each group were used for measurement of brain water content and the other rats were used for biochemical parameters, immunohistochemical and histopathological analyses.

At the end of the experimental period, behavioral analyses were assessed. Then, rats were weighed, anesthetized by sodium thiopental (nesdonal) (I.P., 40 mg kg^−1^), and blood samples were collected by retro-orbital puncture and then centrifuged. The serum was obtained and frozen at − 80 °C for further biochemical analysis. Then rats were sacrificed via cervical dislocation. The liver was excised, rinsed in ice-cold phosphate-buffered saline (PBS) and weighed for calculation of liver/body weight index. The right lobe of the liver was fixed in a 10% phosphate buffered formalin solution for histopathological examination. The left lobe of the liver was homogenized in PBS for further biochemical assays. Then, the skull was cracked opened, the brain was extracted, rinsed in ice-cold saline, and weighed immediately for calculation of brain/body weight index. The right hemisphere was homogenized in ice-cold PBS for further biochemical assays and the left hemisphere was fixed in phosphate buffered formalin for histopathological and immunohistochemical examinations.

### Behavioral analysis

The behavioral tests were implemented in the morning. The tests were manually tracked by a qualified observer (either live or via video recording) blinded to the experimental groups on day 23.

#### Rotarod test

This test was conducted to evaluate the motor coordination of rodents. Accelerating rotarod apparatus (Model No. 7750; Ugo Basile) was used according to the method described in previous reports (Vijitruth et al. 2006; El-Marasy et al. 2019). Rats were trained (3 training sessions /day) for 3 successive days at fixed speed of 4 rotations/min (rpm) to achieve a stable performance. Afterward, on the 4th day, the rats were placed on the testing rod and the starting speed was 4 rpm which has been gradually increased to reach 40 rpm over 300 s. At the end of the experimental model, rats were replaced again for 300 s test session and the fall-off time was recorded.

#### Morris water maze test

The Morris water maze test was applied to examine place learning and memory. It employs equipment which composed of black rounded pool (45 cm depth and 150 cm diameter), filled with 30 cm water (temperature 24 ± 2 ◦C). The pool was subdivided into four quarters (N, S, E, and W). A discreet circular platform (diameter of 10 cm) was placed in S quadrant 2 cm below the water surface and maintained in the same quadrant throughout the whole experiment. The rats were trained to leave swimming by jumping onto the platform (4 trials separated by a 5-min pause on day 22 of the experiment) to learn the place location of the platform. The four different quarters (N, S, E, and W) were used during the four trials as starting points. Each trial ended once the rat jumped onto the platform. If a rat cannot find the platform within a time limit of 120 s, it was guided to the platform and left on for 20 s. A testing trial was conducted 24 h after the last training trial. During the testing trial, latency to the 1st jump on the invisible platform (escape latency, sec) was reported by a qualified person (Khalaf et al. 2020).

#### Open field test

This test evaluates anxiety, locomotor activity levels, and exploratory behavior in rats. The open field test was done using an empty square arena (60 × 60 × 40 cm) with a central square area where the rat is placed in. Rat was allowed to freely explore the arena for a 5 min period and was videotaped. The resting time, line crossings, number of rearing and time spent in the central area were assessed as indicators of locomotor activity, exploratory behavior and anxiety (Fisher and Hughes 1996).

### Assessment of liver and brain indices

Liver and brain indices were assessed according to the following formulas:

$Liver\:index\:=\:Liver\:weight\:/Total\:body\:weight\:\times\:100$ (Ali et al. 2016).

$Brain\:index\:=\:Brain\:weight\:/Total\:body\:weight\:\times\:100$ (Liu et al. 2020).

### Measurement of brain water content

Brain water content was determined by wet/dry weight technique (Shahrokhi et al. 2010; Hajipour et al. 2021). The brain was removed from each rat and weighed before and after 48 h of incubation in a 100 °C oven. The brain water content of each rat was calculated using the following formula:

$$\begin{array}{l}\mathrm{Brain}\;\mathrm{water}\;\mathrm{content}\;\%\\=\lbrack\left(\mathrm{wet}\;\mathrm{tissue}\;\mathrm{weight}-\mathrm{dry}\;\mathrm{tissue}\;\mathrm{weight}\right)\\/\mathrm{wet}\;\mathrm{tissue}\;\mathrm{weight}\rbrack\times100\end{array}$$

### Assessed biochemical parameters

Serum was used for the spectrophotometric measurement of liver function tests (alanine aminotransferase (ALT) and aspartate aminotransferase (AST)) (Human, Wiesbaden, Germany). Serum and brain homogenates were used for measurement of ammonia concentrations (BioVision, USA). The liver and brain homogenates were used for spectrophotometric measurement of malondialdehyde (MDA) and reduced glutathione (GSH) contents (Bio-diagnostic Company, Giza, Egypt) and for ELISA measurement of IL-1β level (Cloud-Clone Corp, USA), according to the manufacturer instructions. Moreover, the levels of MMP-9 and occludin were measured in the brain tissue using commercially available ELISA kits (Cloud-Clone Corp, USA) and (elabscience, USA), respectively, according to the manufacturer instructions.

### Immunohistochemical and histopathological analyses

Formalin fixed liver section and right frontal cortex section from left brain hemisphere were embedded in paraffin wax and sliced (4 to 5 μm) in thickness for staining with hematoxylin and eosin (H & E) to assess structural changes. Three sections were used from each animal with 8 μm between each two sections. Five sampling windows were used in each section sized 100 µm^2^. The severity of histopathological changes in both liver and brain were quantified on a scale from 0 to 3. In liver sections, the scores were assessed according to the extent of degeneration, necrosis, and inflammation. In brain sections, the scores were determined according to the extent of apoptosis, astrocytes swelling, necrosis, and congestion of blood vessels.

- Score 0: no changes,
- Score 1: mild changes; changes affecting ˂25% of the field,
- Score 2: moderate change; changes affecting 25–75% of the field,
- Score 3: severe change; changes affecting ˃75% of the field.

Brain occludin, glial fibrillary acidic protein (GFAP), AQP4 and TLR4 were assessed via immunohistochemistry technique. Briefly, formalin fixed brain slices from different groups were dewaxed and incubated at 4 ◦C overnight with primary antibodies against occludin (Biospes, Chongqing, China, Cat. No. YPA1281, dilution 1/100), GFAP (ServiceBio, Wuhan, China, Cat. No. GB11096, dilution 1/1000), AQP4 (ServiceBio, Wuhan, China, Cat. No. GB11529, dilution 1/600) and TLR4 (ServiceBio, Wuhan, China, Cat. No. GB12186, dilution 1/500). After washing, the samples were incubated with a species-matched secondary antibody for 60 min and visualized with diaminobenzidine. The percentage of stained area was determined using Image J software (NIH, USA) and will be further used for statistical analysis.

### Statistical analysis

One-way analysis of variance (ANOVA) followed by Tukey’s Post-hoc test was used for analysis of parametric data. Kruskal–Wallis test followed by Dunn’s post hoc test were used for analysis of non-parametric data. Statistical analyses and Graphs were carried out using GraphPad Prism version 8.0.0 (Graph Pad Software Inc., SanDiego, USA). Data were expressed as the mean ± standard error of the mean (SEM). p values less than 0.05 were statistically significant.

## Results

The diacerein control group revealed non-significant differences in behavioral tests, brain water content, liver/body weight index, brain/body weight index, liver enzymes, serum and brain ammonia concentrations and both liver and brain histopathological lesions when compared with normal group.

### Effect of diacerein on behavioral tests

**Fig. 2:**
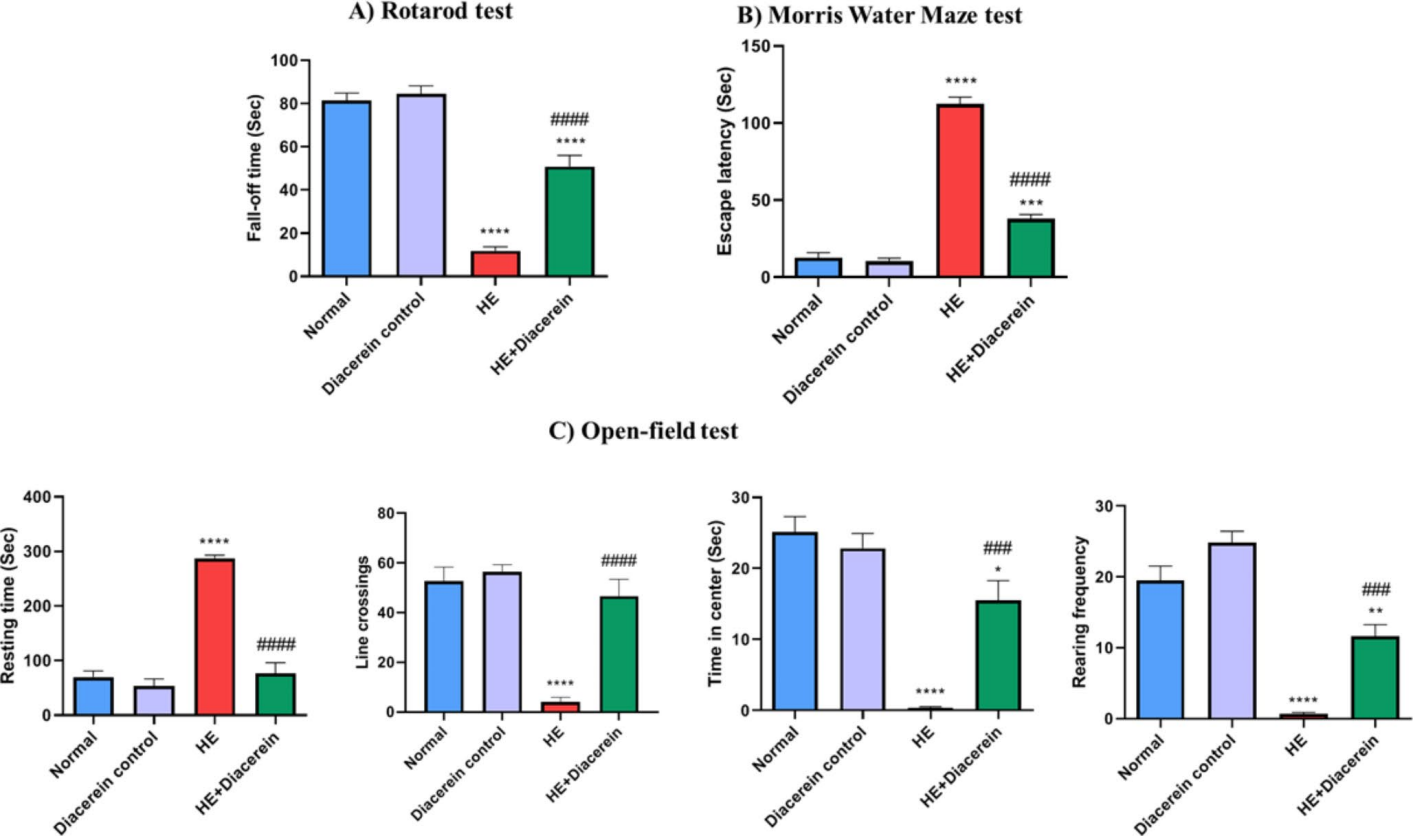
Effect of diacerein on behavioral tests. **(A)** Rotarod test. **(B)** Morris water maze test. **(C)** Open field test. Open field test includes resting time, line crossings, rearing frequency and time spent in center. HE: Hepatic encephalopathy. Values are expressed as mean ± SEM. Statistical analysis was performed using One-Way ANOVA followed by Tukey-Kramer’s test (*n* = 6). ^*^ Significantly different versus normal group at *P* < 0.05. ^**^ Significantly different versus normal group at *P* < 0.01. ^***^ Significantly different versus normal group at *P* < 0.001. ^****^ Significantly different versus normal group at *P* < 0.0001. ^###^ Significantly different versus HE group at *P* < 0.001. ^####^ Significantly different versus HE group at *P* < 0.0001

In rotarod test, HE group showed a marked decrease in fall-off time, compared with normal group (*P* < 0.0001), indicating motor incoordination. While oral administration of 50 mg/kg diacerein ameliorate motor incoordination as it significantly increased fall-off time, compared with HE group (*P* < 0.0001) (Fig. 2A), F (3, 20) = 79.66, *P* < 0.0001.

Moreover, escape latency in Morris water maze test significantly increased in HE group, compared to normal rats (*P* < 0.0001). Treatment with diacerein was accompanied by improvement in learning and memory in HE-rats as noted by decrease in escape latency, compared with HE group (*P* < 0.0001) **(**Fig. 2B), F (3, 20) = 201.9, *P* < 0.0001.

During open field test, HE group showed a significant elevation in resting time (*P* < 0.0001) and a substantial decrease in rearing frequency (*P* < 0.0001), line crossings (*P* < 0.0001) and time spent in center (*P* < 0.0001) as compared to normal group, indicating increased anxiety, impairment in exploratory behavior and locomotor activity. Diacerein treatment relieved anxiety and enhanced exploratory behavior as well as locomotor activity as implied by a significant diminution in resting time (*P* < 0.0001) and a marked increase in rearing frequency (*P* < 0.001), line crossings (*P* < 0.0001) and time spent in center (*P* < 0.001) as compared to HE rats (Fig. 2C). Resting time (F (3, 20) = 66.38, *P* < 0.0001), rearing frequency (F (3, 20) = 47.76, *P* < 0.0001), line crossings (F (3, 20) = 25.90, *P* < 0.0001) and time spent in center (F (3, 20) = 29.73, *P* < 0.0001).

### Effect of diacerein on brain water content

**Fig. 3:**
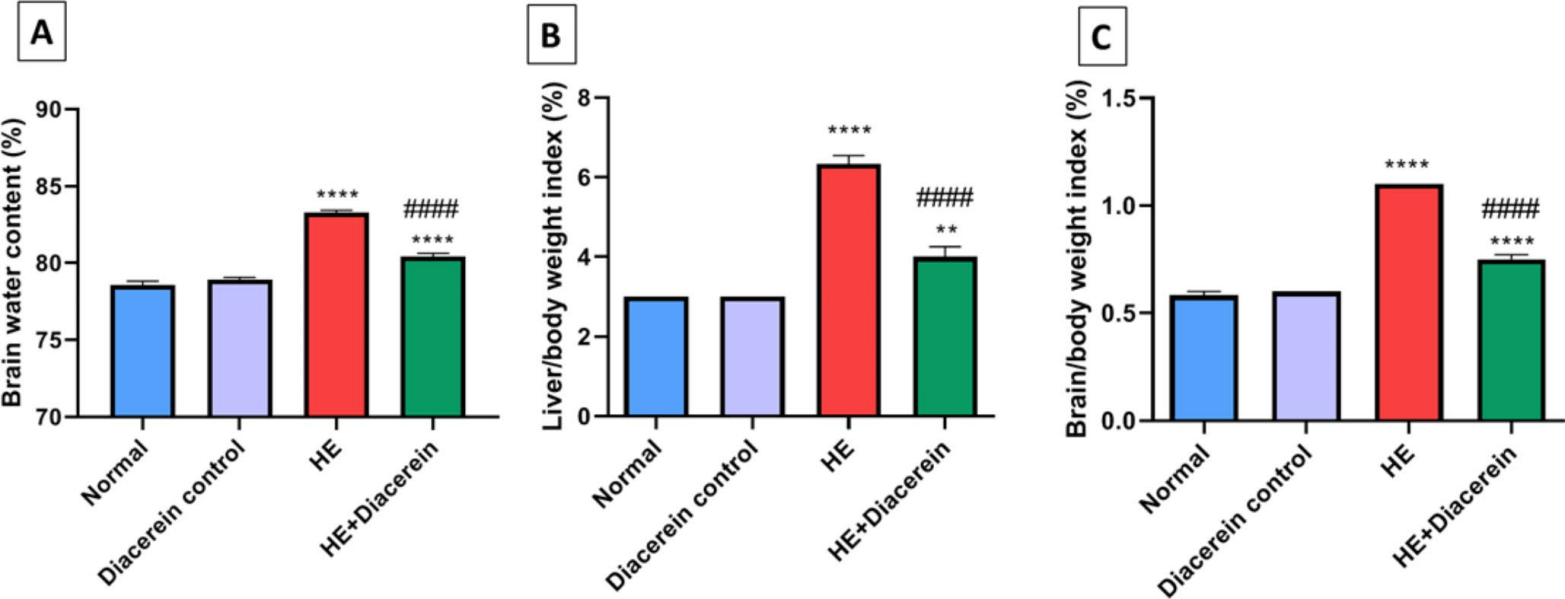
Effect of diacerein on brain water content, liver/body weight and brain/body weight indices. **(A)** Brain water content. **(B)** liver/body weight index. **(C)** Brain/body weight index. HE: Hepatic encephalopathy. Values are expressed as mean ± SEM. Statistical analysis was performed using One-Way ANOVA followed by Tukey-Kramer’s test (*n* = 6). ^**^ Significantly different versus normal group at *P* < 0.01. ^****^ Significantly different versus normal group at *P* < 0.0001. ^####^ Significantly different versus HE group at *P* < 0.0001

A significant elevation in brain water content was observed in HE group in comparison with normal group (*P* < 0.0001). However, diacerein treated group showed a significant reduction in brain water content as compared to HE group (*P* < 0.0001), (Fig. 3A), F (3, 20) = 106.1, *P* < 0.0001.

### Effect of diacerein on liver/body weight and brain/body weight indices

As shown in (Fig. 3B and C), HE group revealed a significant elevation in liver/body weight index, compared with normal group (*P* < 0.0001). Moreover, brain/body weight index markedly increased in HE group compared with normal group (*P* < 0.0001). However, treating rats with 50 mg/kg diacerein showed a significant decrease in both indices compared with HE group (*P* < 0.0001). liver/body weight index (F (3, 20) = 89, *P* < 0.0001) and brain/body weight index (F (4, 25) = 295.7, *P* < 0.0001).

### Effect of diacerein on liver enzymes, serum and brain ammonia concentrations

HE group showed 5.2- and 4-folds (*P* < 0.0001) significant elevation in serum ALT and AST activities, respectively as well as 6.9- and 4.1-folds (*P* < 0.0001) marked increase in serum and brain ammonia concentrations in comparison with normal rats. However, treating rats with diacerein showed a marked reduction in serum ALT, AST activities, serum and brain ammonia concentrations by approximately 2.7-, 1.9-, 3.4- and 2.4-folds (*P* < 0.0001) respectively compared with HE group (Table 1). ALT (F (3, 20) = 1092, *P* < 0.0001), AST (F (3, 20) = 482.4, *P* < 0.0001), serum ammonia (F (3, 20) = 1307, *P* < 0.0001), brain ammonia (F (3, 20) = 3063, *P* < 0.0001).

**Table 1:** Effect of diacerein on liver enzymes, serum and brain ammonia concentrations

Groups	Normal	Diacerein control	HE	HE + Diacerein
**ALT** (IU/L)	62.5 ± 1.09	47.5 ± 1.77	327 ± 7.37^****^	122.16 ± 1.49 ^*****, #####^
**AST** (IU/L)	152.83 ± 2.49	137.5 ± 1.76	617.67 ± 19.74^****^	316.67 ± 3.6 ^*****, #####^
**Serum ammonia** (µg/dl)	22.67 ± 0.56	21 ± 1.00	156.5 ± 3.3^****^	45.5 ± 0.67 ^*****, #####^
**Brain ammonia** (nmol/g.tissue)	441.2 ± 14.05	478.7 ± 10.91	1799 ± 14.02^****^	756.2 ± 3.91 ^*****, #####^

ALT: Alanine aminotransferase, AST: Aspartate aminotransferase, HE: Hepatic encephalopathy

Values are expressed as mean ± SEM. Statistical analysis was performed using One-Way ANOVA followed by Tukey-Kramer’s test (*n* = 6). ^****^ Significantly different versus normal group at *P* < 0.0001. ^####^ Significantly different versus HE group at *P* < 0.0001

###  Effect of diacerein on histopathological alterations in liver

**Fig. 4:**
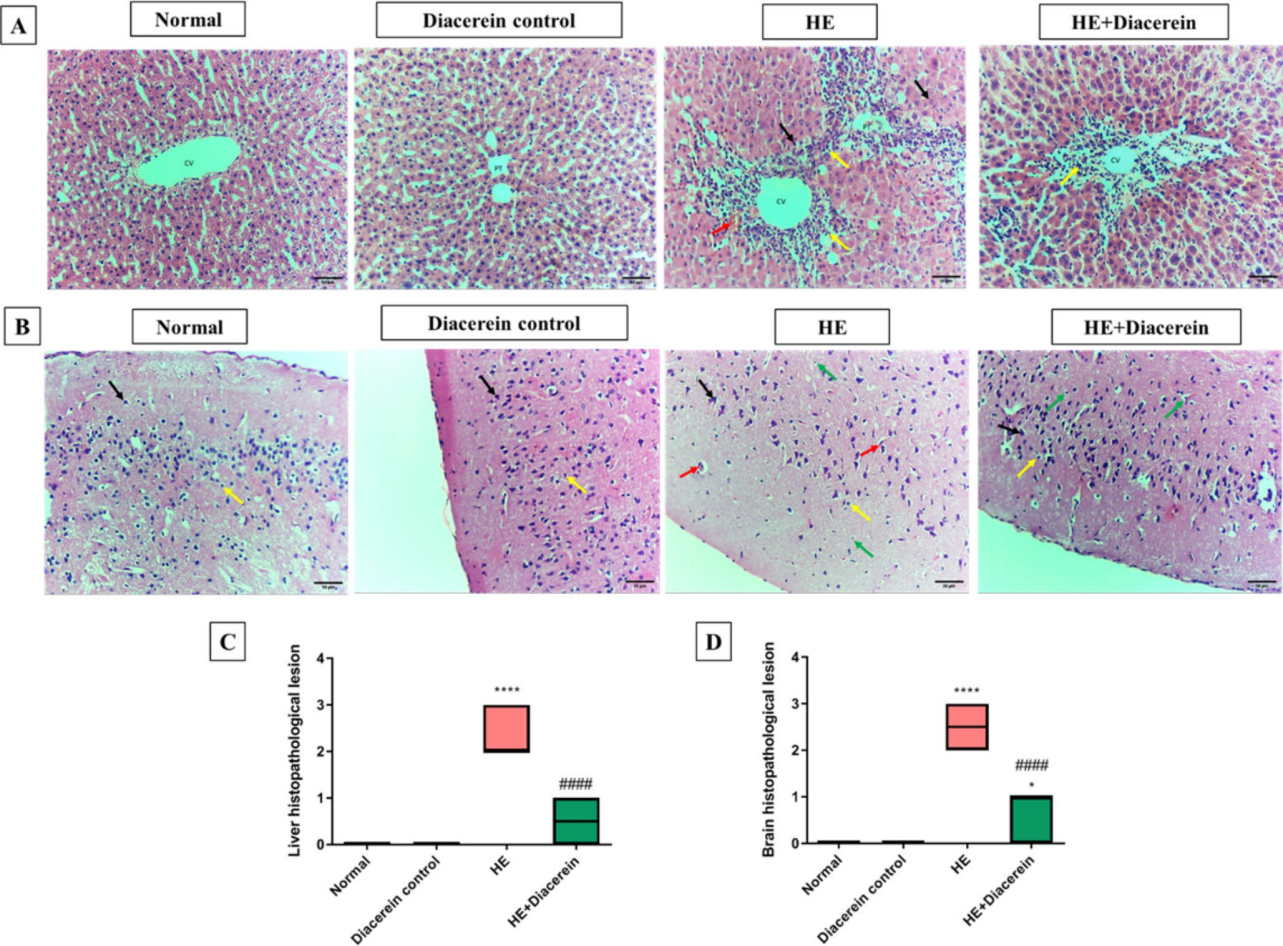
Effect of diacerein on histopathological alterations in liver and brain. **(A)** Histopathological changes of liver sections stained with H&E. CV: Central vein. PT: portal areas. Black arrow indicates disrupted hepatic architecture, centrilobular pyknosis and apoptosis in hepatocytes. Red arrow indicates congestion and hemorrhage. The yellow arrow indicates leukocytic cells infiltration lymphocytes, eosinophils and macrophages. Upper panel (200X, bar 50 μm) and lower panel (400X, bar 20 μm). **(B)** Histopathological changes of brain sections stained with H&E. Black arrow indicates pyramidal neurons. Yellow arrow indicates glial cells. Green arrow indicates shrinkage and apoptosis of pyramidal neurons. Red arrow indicates congestion and hemorrhage. Upper panel (200X, bar 50 μm) and lower panel (400X, bar 20 μm). **(C)** Liver histopathological lesion, scores are expressed as median and interquartile range. (*n* = 6). **(D)** Brain histopathological lesion, scores are expressed as median and interquartile range. (*n* = 6). HE: Hepatic encephalopathy. Statistical analysis was performed using Kruskal–Wallis test followed by Dunn’s post hoc test. ^*^ Significantly different versus normal group at *P* < 0.05. ^****^ Significantly different versus normal group at *P* < 0.0001. ^####^ Significantly different versus HE group at *P* < 0.0001

Histopathological examination of H&E-stained liver tissues revealed that intact liver arrangement of hepatic cords around central vein, normal portal areas and sinusoids with absence of any evidence of degeneration, necrosis, or inflammation in normal group. Rats treated with TAA in HE group showed a marked disrupted hepatic architecture, centrilobular pyknosis and apoptosis in hepatocytes (black arrow), significant congestion and hemorrhage (red arrow) accompanied with leukocytic cells infiltration lymphocytes, eosinophils and macrophages (yellow arrow). However, these findings were significantly ameliorated in the diacerein treated group, which showed improvement in histopathological picture of liver lobules and reduction in inflammation, apoptosis and congestion (Fig. 4A and C).

###  Effect of diacerein on histopathological alterations in brain

In cross-sectional images of brain tissues stained with H&E, Normal group showed normal structure of cerebral cortex, normal pyramidal neurons (black arrow), and glial cells (yellow arrow) with absence of any evidence of necrosis, astrocytes swelling, or inflammation. HE group revealed congestion and hemorrhage (red arrow), marked shrinkage and apoptosis (green arrow) of pyramidal neurons. Contrariwise, HE + Diacerein group showed significant amelioration in histological lesions of cerebral cortex and decreasing in inflammation, apoptosis, and congestion **(**Fig. 4B and D**)**.

###  Effect of diacerein on TLR4 expression in brain

**Fig. 5:**
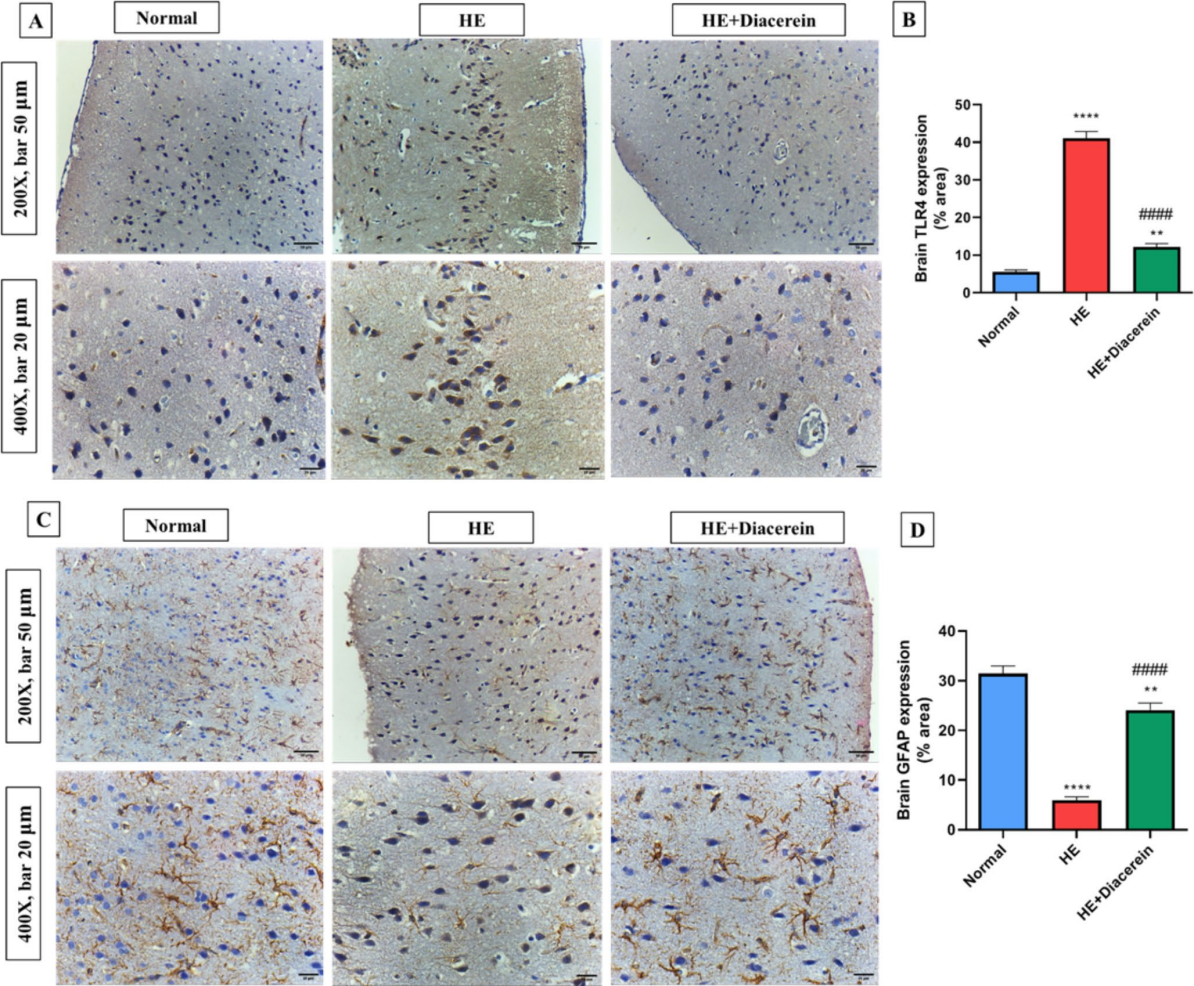
Effect of diacerein on TLR4 and GFAP expressions in brain. **A**,** B)** The expression of TLR4 using immunohistochemistry, Bars represent the % area in brain sections stained with anti-TLR4 antibodies, Upper panel (200X, bar 50 μm) and lower panel (400X, bar 20 μm). **C**,** D)** The expression of GFAP using immunohistochemistry, Bars represent the % area in brain sections stained with anti-GFAP antibodies, Upper panel (200X, bar 50 μm) and lower panel (400X, bar 20 μm). HE: Hepatic encephalopathy. Values are expressed as mean ± SEM. Statistical analysis was performed using One-Way ANOVA followed by Tukey-Kramer’s test (*n* = 6). ^**^ Significantly different versus normal group at *P* < 0.01. ^****^ Significantly different versus normal group at *P* < 0.0001. ^####^ Significantly different versus HE group at *P* < 0.0001

As shown in (Fig. 5A and B), examination of immunostained brain sections from HE group showed a significant elevation in TLR4 expression by about 7.4-folds (*P* < 0.0001), compared with normal group. Meanwhile, brain sections from HE + Diacerein group showed a notable decrease in the expression of TLR4 by 3.4-folds (*P* < 0.0001) as compared to HE group, F (2, 15) = 234.8, *P* < 0.0001.

###  Effect of diacerein on GFAP expression in brain

Immunostained sections from brain tissues revealed a significant reduction in GFAP expression by approximately 81% (*P* < 0.0001) in HE group as compared to normal group. However, brain specimens from diacerein treated group showed a marked surge in percentage area immunostained with GFAP by nearly 4-folds (*P* < 0.0001), compared with HE group (Fig. 5C and D), F (2, 15) = 108.4, *P* < 0.0001.

###  Effect of diacerein on oxidative stress and inflammation in liver and brain

**Fig. 6:**
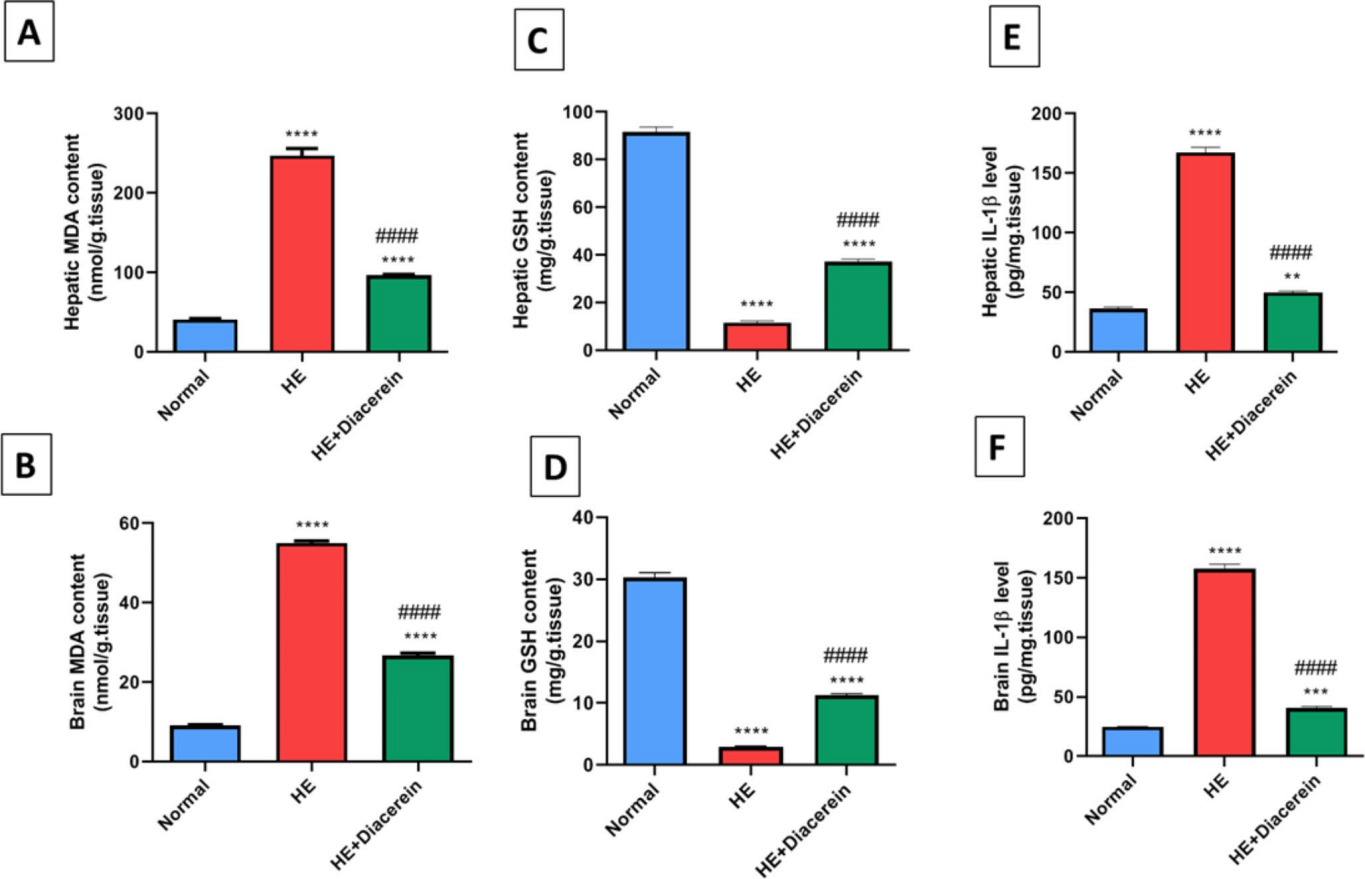
Effect of diacerein on oxidative stress and inflammation in liver and brain. **(A)** Hepatic MDA content. **(B)** Brain MDA content; **(C)** Hepatic GSH content. **(D)** Brain GSH content. **(E)** Hepatic IL-1β level. **(F)** Brain IL-1β level. HE: Hepatic encephalopathy

HE group revealed 6- and 6.1-folds (*P* < 0.0001) marked elevation in hepatic MDA and brain MDA contents, respectively and accompanied with 7.8- and 10.4-folds (*P* < 0.0001) significant reduction in hepatic GSH and brain GSH contents, respectively, in comparison with normal group. Surprisingly, diacerein treatment exhibited a marked reduction in both hepatic and brain MDA contents as well as a significant increment in GSH contents in liver and brain, as compared to HE rats (*P* < 0.0001) (Fig. 6A, B, C and D). Hepatic MDA (F (2, 15) = 413.9, *P* < 0.0001), brain MDA (F (2, 15) = 2148, *P* < 0.0001), hepatic GSH (F (2, 15) = 773.4, *P* < 0.0001) and brain GSH (F (4, 25) = 773.6, *P* < 0.0001).

Values are expressed as mean ± SEM. Statistical analysis was performed using One-Way ANOVA followed by Tukey-Kramer’s test (*n* = 6). ^**^ Significantly different versus normal group at *P* < 0.01. ^***^ Significantly different versus normal group at *P* < 0.001. ^****^ Significantly different versus normal group at *P* < 0.0001. ^####^ Significantly different versus HE group at *P* < 0.0001.

As shown in (Fig. 6E and F), Hepatic and brain IL-1β level were significantly elevated in HE group by 4.6- and 6.3-folds (*P* < 0.0001), respectively as compared to normal group. On the other hand, diacerein administration caused a significant diminution in IL-1β level in liver and brain by 3.4- and 3.9-folds (*P* < 0.0001), respectively as compared to HE group. Hepatic IL-1β (F (2, 15) = 698.1, *P* < 0.0001) and brain IL-1β (F (2, 15) = 966.9, *P* < 0.0001).

### Effect of diacerein on MMP-9 level in brain

**Fig. 7:**
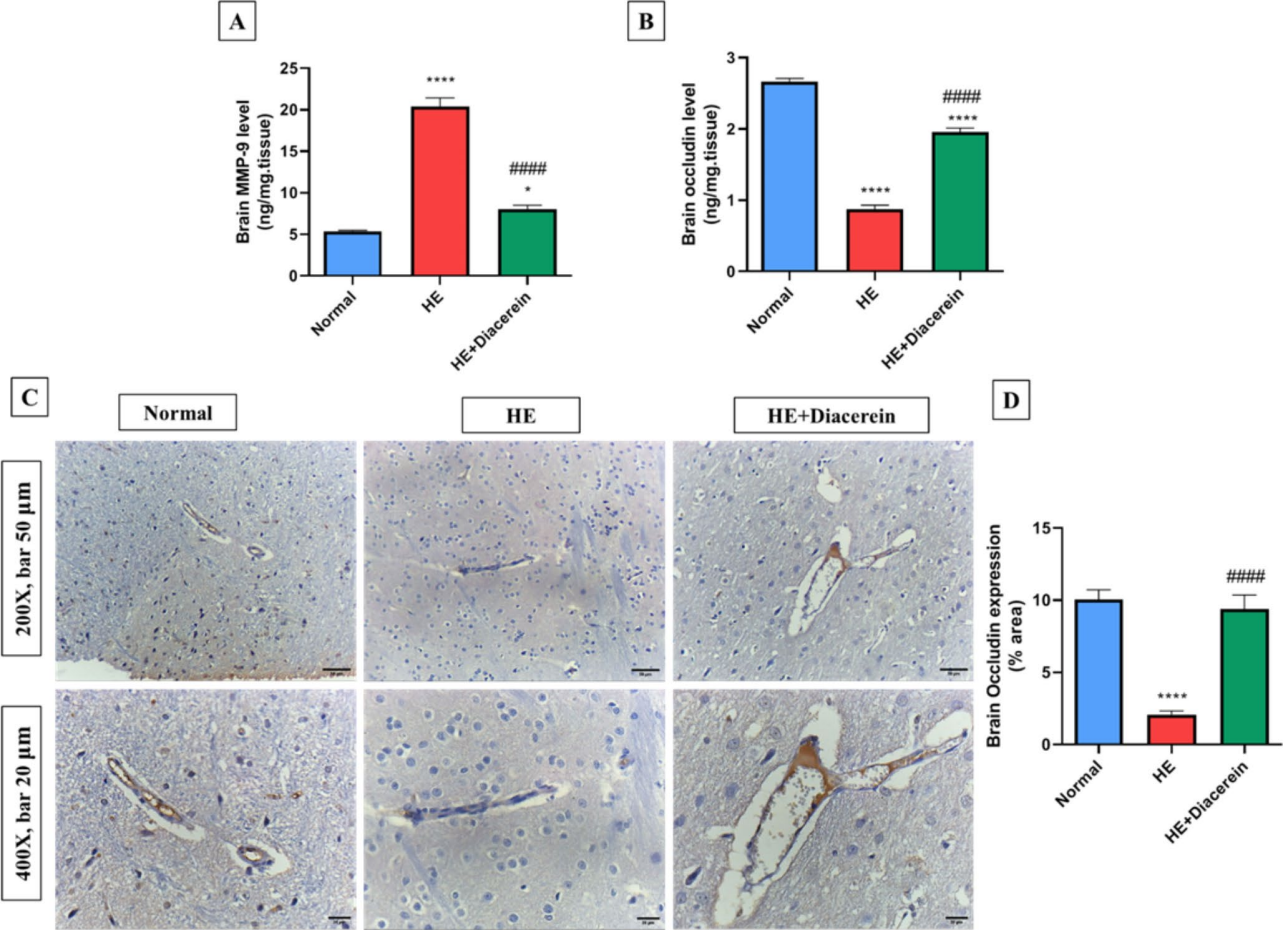
Effect of diacerein on MMP-9 level, occludin level and expression in brain. **(A)** Brain MMP-9 level. **(B)** Brain occludin level using ELISA. **C**,** D)** The expression of occludin using immunohistochemistry, Bars represent the % area in brain sections stained with anti-occludin antibodies, Upper panel (200X, bar 50 μm) and lower panel (400X, bar 20 μm). HE: Hepatic encephalopathy. Values are expressed as mean ± SEM. Statistical analysis was performed using One-Way ANOVA followed by Tukey-Kramer’s test (*n* = 6). ^*^ Significantly different versus normal group at *P* < 0.05. ^****^ Significantly different versus normal group at *P* < 0.0001. ^####^ Significantly different versus HE group at *P* < 0.0001

As shown in (Fig. 7A), remarkable increase in MMP-9 level in brain was detected in HE group as compared to normal group (*P* < 0.0001). After 21 days of daily oral administration of diacerein, MMP-9 level in brain was decreased significantly by about 60.7% (*P* < 0.0001) in comparison with HE group, F (2, 15) = 139.5, *P* < 0.0001.

### Effect of diacerein on occludin level and expression in brain

TAA administration in HE group caused a significant decrease in brain occludin level by 3-folds (*P* < 0.0001), compared with normal group. Conversely, HE + Diacerein group showed a remarkable increase in occludin level in brain by 2.2-folds (*P* < 0.0001), compared with HE group (Fig. 7B), F (2, 15) = 255, *P* < 0.0001.

Immunohistochemical analysis of brain tissues exhibited a marked reduction of occludin positive staining in HE group by about 79.2% (*P* < 0.0001) as compared to normal group, while treatment with diacerein 50 mg/kg revealed a significant increase in occludin expression in brain in comparison with HE group (*P* < 0.0001) (Fig. 7C and D), F (2, 15) = 39.1, *P* < 0.0001.

### Effect of diacerein on AQP4 expression in brain

**Fig. 8:**
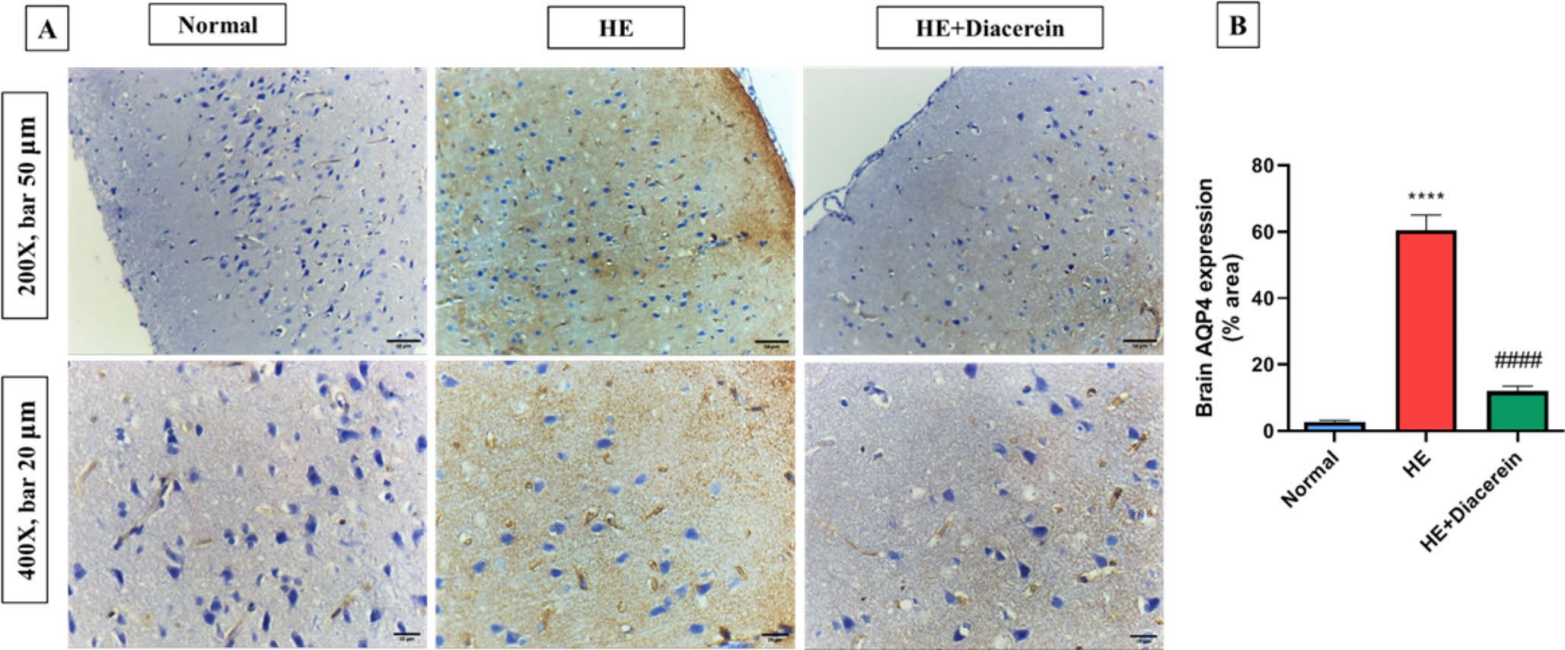
Effect of diacerein on AQP4 expression in brain. **A**,** B)** The expression of AQP4 using immunohistochemistry, Bars represent the % area in brain sections stained with anti-AQP4 antibodies, Upper panel (200X, bar 50 μm) and lower panel (400X, bar 20 μm). HE: Hepatic encephalopathy. Values are expressed as mean ± SEM. Statistical analysis was performed using One-Way ANOVA followed by Tukey-Kramer’s test (*n* = 6). ^****^ Significantly different versus normal group at *P* < 0.0001. ^####^ Significantly different versus HE group at *P* < 0.0001

As shown in (Fig. 8A and B), TAA-induced HE in rats displayed significant elevation in % area immunostained with AQP4 in brain by nearly 21.7-folds (*P* < 0.0001) as compared to normal group. Interestingly, administration of diacerein caused a remarkable decrease in brain AQP4 expression by approximately 5-folds (*P* < 0.0001) as compared to HE group, F (2, 15) = 118.8, *P* < 0.0001.

## Discussion

The current study provides new perspective on the neuroprotective effect of diacerein (50 mg/kg) through modulation of TLR4/AQP4/MMP-9 pathway in TAA-induced HE rat model. Diacerein significantly relieves motor and cognitive deficits, cerebral and hepatic histopathological changes, oxidative stress, hepatic function, hyperammonemia and cerebral edema in HE rats.

Diacerein is a well-known anthraquinone derivative which is clinically used to treat osteoarthritis. Its therapeutic effect is related to its anti-inflammatory and antioxidant activities which recently has been shown to relieve testicular injury, nephrotoxicity, and hepatotoxicity (Almezgagi et al. 2020; Mohamed Kamel et al. 2022). The current study offers a new perspective neuroprotective effect of diacerein against TAA-induced HE in rats through investigating its protective impact on liver injury, hyperammonemia, cognitive and motor deficits, astrocyte swelling and BBB integrity.

In the current study, I.P. injection of TAA at a dose of 200 mg/kg for three alternative days is a well standardized and validated method for the induction of acute liver failure and subsequent HE (Abdelaziz et al. 2015; Khodir and Said 2020). TAA induced liver damage was proofed by significant increased serum ALT and AST activities which indicates leakage and damage of hepatocyte’s cell membrane integrity and function (Anbarasu et al. 2012; Farjam et al., 2012). Moreover, TAA induces hepatocyte’s inflammation and oxidative stress as evidenced by significant increase in hepatic IL-1β level and hepatic MDA content along with significant decrease in hepatic GSH content, compared to normal group. Also, liver/body weight index was markedly increased in TAA treated rats when compared to normal rats, which indicates the incidence of acute liver failure, in parallel with former report (Khodir and Said 2020). Furthermore, histopathological examination of liver tissues revealed marked disrupted hepatic architecture, apoptosis, hemorrhage, and leukocytic cells infiltration, in agreement with previous studies (Khodir and Said 2020; de David et al. 2011).

Taken together, damaged hepatocytes can’t adequately eliminate gut-derived ammonia, as a result, ammonia enters the systemic circulation, and eventually the brain, where it exerts detrimental HE effects (Jayakumar and Norenberg 2018). In our study, TAA-induced HE rats showed significant elevation in serum and brain ammonia concentrations in comparison with normal rats, in consistent with the previous reports (Khodir and Said 2020; Saleh et al. 2021).

Elevation of ammonia level was considered a key factor associated with HE- induced neurological disorders (El-Marasy et al. 2019; Heidari et al. 2016; Butterworth et al. 1987). In the present study, TAA-induced HE rats showed marked decline in the fall-off time in rotarod test and significant raise in the escape latency in Morris water maze test which reflects the impairment in motor coordination and memory, respectively, in agreement with the previous reports (El-Marasy et al. 2019; Jamshidzadeh et al. 2017). Besides, rats treated with TAA suffered from anxiety, impairment in exploratory behavior and locomotor activity as proved by significant decrease in the time spent in the central zone, line crossings and number of rearing along with significant increase in resting time in open field test as compared with the normal group, in agreement with the previous studies (Jamshidzadeh et al. 2017; Ferah Okkay et al. 2022).

In fact, the state of hyperammonemia in TAA treated rats were also associated with marked neuronal histopathological changes. Brain tissues of TAA-treated rats revealed congestion, hemorrhage, apoptosis, and astrocytes swelling. Moreover, brain water content and brain/body weight index were markedly increased in TAA-induced HE rats as compared with normal rats. In agreement, HE-induced astrocyte swelling, and subsequent cerebral edema has been reported to be the initial step of brain injury. The progression of acute liver failure increased the risk of brain edema which may progress to brain herniation; a main cause of death (Perazzo et al. 2012).

Oral administration of diacerein ameliorated TAA-induced liver injury and function as proofed by marked reduction in serum ALT and AST activities along with significant lowering of liver/body weight index and marked improvement in the histopathological features of liver lobules as reduction in inflammation and congestion, compared to HE group. Moreover, diacerein relieved hepatic inflammation and oxidative stress as evidenced by significant decrease in hepatic IL-1β level and hepatic MDA content along with significant increase in hepatic GSH content, compared to HE group. The hepatoprotective effect of diacerein was previously discussed in different animal models (Ibrahim et al. 2021; Bu et al. 2018; Wang et al. 2022; Abdelfattah et al. 2023).

Diacerein-treated rats showed improvement in motor coordination and memory in HE-rats as evidenced by significant increase in the fall-off time in rotarod test and significant decrease in the escape latency in Morris water maze test, respectively. Also, diacerein ameliorates anxiety, impairment in exploratory behavior and locomotor activity in HE rats as noted by significant elevation in the time spent in the center, line crossings and number of rearing along with significant lowering in resting time in open field test. This enhancement of motor and cognitive deficits may related to the effect of diacerein on lowering hyperammonemia, brain water content and brain/body weight index along with marked amelioration in brain histopathological changes. The neuroprotective effect of diacerein was reported in various animal models (Sharma and Mishra 2023; Karvat et al. 2023).

As we have mentioned before, brain edema is a serious consequence of persistent hyperammonemia in which if inadequately controlled it will lead to brain death. One of the main causes of brain edema is astrocytes swelling. Astrocytes account for about one-third brain volume and play a crucial role in the homeostasis of the central nervous system. They contribute to formation and maintenance of the BBB, supplying energy for the neurons, neurotransmission, and regulation of metabolic and synaptic plasticity (Sepehrinezhad et al. 2020).

One of the main constituents of astrocytes is GFAP; an astrocyte intermediate filament and contributes in the regulation of astrocyte’s motility and morphology along with structural stability of astrocyte processes (Pekny and Wilhelmsson 2006). GFAP is considered as a marker of mature astrocytes (Li et al. 2020). A previous in vitro study revealed that exposure of cultured astrocytes to ammonia resulted in a significant decrease in GFAP gene expression along with cellular swelling, it suggested that loss of GFAP may alter the visco-elastic properties of the astrocyte’ membrane inducing cellular swelling and cerebral edema (Norenberg et al. 1990). Another study revealed loss of GFAP expression in postmortem cerebral tissues from HE patients who died as a result of brain herniation due to acute liver failure (Thumburu et al. 2014). In a harmony with the previous reports, our results showed a significant reduction in GFAP expression in immunostained brain sections of HE group as compared to normal group.

The AQP4, is a water channel protein present in astrocytic foot processes that play a crucial role in cerebral water regulation (Badaut et al. 2011; Fukuda et al. 2012). In agreement with the previous reports, the current study showed a significant upregulation of AQP4 protein expression in brain tissues of HE group when compared to normal group, which correlates with HE astrocytes swelling and hence cerebral edema (Rama Rao et al. 2010; Margulies et al. 1999). It was reported that ammonia- treated cultured astrocytes revealed a significant increase in the AQP4 protein expression as well as astrocytes swelling, suggesting the role of AQP4 channels in astrocytic swelling in hyperammonemia (Rama Rao et al. 2003).

Several mechanisms have been suggested to correlate the hyperammonemia and the increase in brain astrocytic AQP4 channels. Firstly, it is well known that oxidative stress represents a cornerstone in the pathogenesis of HE induced by hyperammonemia, as ammonia upregulates cellular free radicals and downregulates the endogenous antioxidants (Norenberg et al. 2004). It was reported that, oxidative stress upregulates AQP4 protein expression in cultured astrocyte (Arima et al. 2003). On the other hand, antioxidant-treated astrocytic cultures revealed a significant downregulation of AQP4 and astrocyte swelling (Jayakumar et al. 2006). As showed in our study, HE group revealed significant elevation in brain MDA contents accompanied with significant lowering in brain GSH content. Secondly, hyperammonemia-induced neuro-inflammation/ microglia activation may also upregulate astrocytic AQP4 protein expression in the brain and subsequent cellular edema (Ohnishi et al. 2014). It was reported that, elevated microglia activation markers as IL-1β, accelerated the onset of encephalopathy while deletion of the IL-1β gene attenuated brain edema hence delayed encephalopathy (Bémeur et al. 2010). In accordance, our results revealed a marked increase in brain IL-1β level in HE rats compared to normal rats. Together, oxidative stress and microglia activation induced by hyperammonemia promote upregulation of brain TLR4 and further astrocyte swelling, in a harmony with our results (Jayakumar et al. 2014). In another study, acetaminophen does not produce brain edema in TLR-4 knock-out mice (Shah et al. 2013). Moreover, a recent study showed that attenuating TLR4 in TAA- induced HE model ameliorates neuro-inflammation, oxidative stress, astrocyte swelling and hence cerebral edema (Lu et al. 2020). In the present study, diacerein relieves astrocyte swelling and subsequent brain edema as proved by significant elevation in brain GFAP protein expression and brain GSH content along with downregulation of brain AQP4 and TLR4 proteins expression, brain IL-1β level and brain MDA content, when compared to HE group.

Moreover, recent study revealed that the neuro-inflammation altered BBB permeability via upregulation of MMP-9 in acute hypobaric hypoxia related encephalopathy model (Liu et al. 2022). MMP-9 has been involved in BBB disruption via digestion of tight junction proteins such as occludin which can result in selective permeability to small molecules like water and ammonia (Chen et al. 2009, 2011). Several hepatic encephalopathy evidences revealed that MMP-9 activation and occludin suppression were contributed to the alterations in BBB permeability and hence brain edema (Skowrońska et al. 2012; Dhanda and Sandhir 2018). In agreement, our results showed a marked increase in brain MMP-9 level along with marked decrease in brain occludin protein level and tissue expression in HE group, compared to normal group. On the flip side, diacerein ameliorates alterations in BBB permeability as proved by significant decrease in brain MMP-9 level, and significant increase in brain occludin protein level and tissue expression, compared to HE rats.

In conclusion, our findings revealed that diacerein has a promising neuroprotective effect and may decrease the progression of HE induced in acute liver failure. The neuroprotective effect of diacerein was proved by improvement of motor and cognitive deficits, brain histopathological changes and hepatic functions; hallmarks of HE. In addition, diacerein ameliorates hyperammonemia induced cerebral edema and maintains BBB integrity through modulation of TLR4/AQP4/MMP-9 axis. To the best of our knowledge, the present study is the first that might shed some light on the neuroprotective effect of diacerein in HE. So, more experimental studies are required to explore more molecular mechanisms that may support the neuroprotective effect of diacerein in HE models.

## Data Availability

No datasets were generated or analysed during the current study.

## Abbreviations

ALT: Alanine aminotransferase
AST: Aspartate aminotransferase
AQP4: Aquaporin 4
GFAP: Glial fibrillary acidic protein
BBB: Blood brain barrier
GSH: Glutathione
H&E: Hematoxylin and eosin
HE: Hepatic encephalopathy
MMP-9: matrix metalloproteinase 9
MDA: Malondialdehyde
TAA: Thioacetamide
TLR4: Toll-like receptor-4
